# Evidence for Concrete but Not Abstract Representation of Length During Spatial Learning in Rats

**DOI:** 10.1037/xan0000044

**Published:** 2014-11-24

**Authors:** Julie R. Dumont, Peter M. Jones, John M. Pearce, Yutaka Kosaki

**Affiliations:** 1 School of Psychology, Cardiff University

**Keywords:** spatial learning, stimulus generalization, magnitude discrimination

## Abstract

In 4 experiments, rats had to discriminate between the lengths of 2 objects of the same color, black or white, before a test trial with the same objects but of opposite color. The experiments took place in a pool from which rats had to escape by swimming to 1 of 2 submerged platforms. For Experiments 1 and 2, the platforms were situated near the centers of panels of 1 length, but not another, that were pasted onto the gray walls of a square arena. The acquired preference for the correct length was eliminated by changing the color of the panels. In Experiment 3, the platforms were situated near the middle of the long walls of a rectangular pool, and in Experiment 4 they were situated in 1 pair of diagonally opposite corners of the same pool. Changing the color of the walls markedly disrupted the effects of the original training in both experiments. The results indicate that rats represent the length of objects not by their abstract, geometric attributes but in a more concrete fashion such as by a mental snapshot or by the amount of color stimulation they provide.

A wide variety of species use cues provided by the shape of their environment in order to reach a hidden goal. Thus the ability to find a goal located in one corner of a rectangular arena has been demonstrated in ants (Wystrach, Cheng, Sosa, & Beugnon, 2011), chicks (Tommasi & Vallortigara, 2000), rats (Cheng, 1986), monkeys (Gouteux, Thinus-Blanc, & Vauclair, 2001), pigeons (Kelly, Spetch, & Heth, 1998), fish (Sovrano, Bisazza, & Vallortigara, 2002), infant humans (Wang, Hermer, & Spelke, 1999), and adult humans (Hermer-Vazquez, Spelke, & Katsnelson, 1999). An obvious question raised by these findings concerns the manner in which the shape of the environment is used to indicate where a goal can be found. According to Gallistel (1990), exposure to any environment will result in an abstract representation that encodes information about its shape. Moreover, this representation is said to be encoded in a geometric module that is impenetrable to nongeometric cues and based exclusively on relative positions of points within the shape (see Gallistel, 1990, pp. 209–212). Although this conclusion concerning the impenetrability of the geometric module was based on computational considerations, it can also be justified by evolutionary principles. As Gallistel points out, if a nutcracker should hide food during warm weather, but must retrieve it during winter when the ground is covered with snow, then many of the landmarks that might have been used to identify where the food was buried will no longer be visible. The covering of snow, however, will have little impact on information provided by the shape of the environment, and if this information was referred to during caching then it will still be available to guide the bird when it returns in winter to find its hidden supply of food.

A similar view concerning the representation of geometric information has been expressed by Cheng (1986, see pp. 172–173), who argued that animals possess a metric frame that encodes geometric information, including the shape of the environment. Although this frame allows animals to identify where specific objects can be found, relative to the shape of the environment, like Gallistel, Cheng assumes that information about the shape of the environment is encoded in an abstract form, independently of the nongeometric, physical characteristics of the objects creating the shape. It is not just shape information that is encoded in this abstract manner, the geometric module and the metric frame are assumed to be responsible for encoding any geometric properties of the environment to which the animal is sensitive.

As the example of the nutcracker makes clear, an obvious implication of the foregoing proposals is that once an animal has been trained to find a goal with reference to the geometric properties of the environment, changing the physical characteristics of those properties should not impair the ability of the animal to find the goal. There is rather little evidence that relates to this prediction, and that which exists is hard to interpret. One reason why the experiments are hard to interpret is that, with the possible exception of a study by Cheng (1986), none of them was designed with the intention of testing the above proposals of Gallistel (1990) and Cheng. Instead, they were more concerned with exploring the interaction between spatial learning based on geometric and nongeometric cues.

Gray, Bloomfield, Ferrey, Spetch, and Sturdy (2005) trained chickadees to find food in one corner of a rectangular arena. For one group, training took place in a rectangle with a long blue wall and three white walls and with food located in a corner created by two white walls. During a test in an all-white rectangle, there was no hint that performance was disrupted by the removal of the blue wall. Although this pattern of results is consistent with the proposals of Gallistel (1990) and Cheng (1986), it is possible that the birds paid little attention to the blue wall. Rather than construct a global representation of the overall shape of the arena, they may have constructed a more local geometric representation based on the corner where the food was situated—for example, they may have identified the correct corner as the one where, say, a short white wall was to the left of a long white wall. There is certainly evidence that rats (Gilroy & Pearce, 2014; McGregor, Jones, Good, & Pearce, 2006; Pearce, Good, Jones, & McGregor, 2004) and chicks (Tommasi & Polli, 2004) make use of local cues when navigating in an arena with a distinctive shape. In keeping with this suggestion, a second group of chickadees was trained to find food in a corner created by a blue wall and a white wall in the same rectangle, before being tested in an all-white rectangle. On the basis of what has just been said, this group should identify the correct corner as one where, say, a blue wall was to the left of a white wall, and thus be unable to differentiate between the correct and incorrect corners during the test in the all-white rectangle. The results confirmed this prediction and, at the same time, pose a challenge to the proposals of Cheng and Gallistel. According to these proposals the change to the apparatus for the test should have exerted no influence on searching for the goal.

More encouraging support for the claim that geometric information is processed in an abstract manner can be found in an experiment by Pearce, Graham, Good, Jones, and McGregor (2006). Two groups of rats were trained to swim to a submerged platform in one corner of a rectangular pool. For one group, the long walls were black and the short walls white on half the trials, while on the other trials, the long walls were white and the short walls were black. For another group, the four walls were always white. A subsequent test trial in a pool with four white walls revealed that both groups preferred the geometrically correct over the incorrect corners. Thus in keeping with the proposals of Cheng (1986) and Gallistel (1990), a change to the color of the arena for the test trial did not disrupt navigation with reference to cues provided by its shape. The change to the appearance of the arena was not complete for the test trial, however, and rats may have relied on those cues that were present in both stages in order to identify where to search. For example, the first group may have identified the correct corner as being at a certain end of a long black wall or a long white wall. By relying on the last piece of information it would then be able to find the goal successfully in the test trial. The experiment by Pearce et al. (2006), therefore, provides, at best, equivocal support for the proposals of Cheng and Gallistel.

The foregoing alternatives to the proposals of Cheng (1986) and Gallistel (1990), as explanations put for the above results, are not applicable to an experiment by Cheng (1986), who trained rats to find food in a black and white corner of an arena with one white wall and three black walls. When they were tested in an arena with four black walls, the rats showed a strong preference for searching in the two geometrically correct corners. Clearly, such behavior would not be expected if rats learned to find food in a black and white corner or at a certain end of a long white wall. It would be expected, however, if rats identified the correct corner with reference to geometric cues whose influence is unaffected by changes to the appearance of the objects on which they are based. Thus, this result is entirely consistent with the proposals of Cheng (1986) and Gallistel (1990), but, once again, it may have occurred for different reasons. Throughout the training phase, as well as during the test trials, distinctive nongeometric landmarks were situated in the two incorrect corners. Rats may then have been reluctant to head toward them during testing and by default preferred to visit the correct rather than incorrect corners.

A problem with all the foregoing findings is that when the concrete properties of a geometric cue were changed, it is hard to be certain that animals relied on this geometric cue when it was found that the change did not affect their ability to find the goal. Instead, they may have relied on some other geometric or nongeometric cue whose appearance was not altered for the test trial. One method for circumventing this problem is to train animals in such a way that only geometric cues can be used to find a goal and to change the concrete characteristics of all of them for the test trial. If the proposals of Gallistel (1990) and Cheng (1986) are correct, then searching for the goal will be unaffected by the change. In fact, the only experiment to adopt this design was conducted by Graham, Good, McGregor, and Pearce (2006). Rats were trained to find a submerged platform in a right-angled corner of a kite-shaped pool. The four walls were entirely black for some rats and entirely white for others during training, whereas for testing, they were either the same color as for training or the opposite color. In contrast to predictions, from Gallistel (1990) and Cheng (1986), the preference for the correct corner was stronger when the walls of the arena for the test trial were the same color, rather than a different color, to that for the training stage. It would, however, be unwise to place too much emphasis on this outcome. It was obtained with a single group, which was included as a control condition in an investigation of potentiation in spatial learning. As a consequence, the sequence of test trials just described was not counterbalanced within the group, and there were also several test trials in the familiar environment before the two test trials just described took place.

The various interpretations that can be applied to each of the above studies, make it unreasonable to draw any firm conclusions from them concerning the claim by Gallistel (1990) and Cheng (1986) that the control by geometric cues over searching for a goal should not be affected by a change to the concrete characteristics of those cues. The purpose of the experiments reported in this paper, therefore, was to provide for the first time a direct test of these proposals. It is evident from the foregoing discussion that when an animal is required to find a goal with reference to the shape of the environment, a variety of geometric cues can be used to aid this search. In the case of a rectangle with a goal hidden in one corner, for example, Pearce et al. (2004) proposed that the goal could be found by searching in a particular location as defined by the overall shape of the environment, or by more local properties such as a particular corner, or at one end of a wall of certain length. In order to simplify the interpretation of the results, therefore, the first two experiments were conducted in an environment where only the length of an object could be used to indicate where a goal is situated. Because length can be regarded as a geometric property, it follows from the proposals of Gallistel (1990) and Cheng (1986) that if a goal can be found with reference to the object’s length, then changing its concrete properties will not impair the capacity of subjects to find the goal. This prediction was tested in Experiments 1 and 2. The final experiments were conducted in order to determine if the conclusions drawn from the first two experiments also apply when the position of the goal is indicated by the shape of the environment.

## Experiment 1

**Figure 1 fig1:**
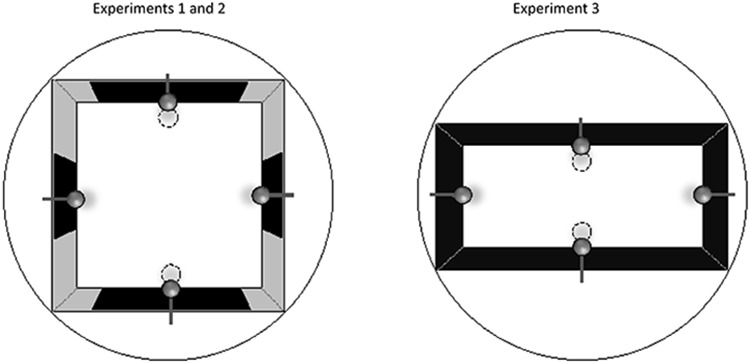
Sketches of the square arena with black panels pasted onto gray walls that was used for Experiments 1 and 2 (left-hand panel), and of the rectangular arena with black walls that was used for Experiment 3 (right-hand panel). Filled circles depict landmarks, which were identical. Dashed circles depict the location of two submerged escape platforms, which were directly beneath a landmark.

The experiment was performed in a square swimming pool with gray walls to which black or white rectangular panels were attached in the middle (see Figure 1). The panels were of the same height, but on two opposite walls, they were long, 100 cm, and on the two remaining walls, they were short, 50 cm. Rats were placed in the pool and required to escape from it by swimming to one of two submerged platforms that were situated beside the middle of the walls containing the long panels. In order to help rats identify where the middle was situated, a landmark was attached to the top, at the center, of each wall. The four landmarks were identical and were thus of no help for distinguishing between long and short panels. Kosaki, Jones, and Pearce (2013) have shown that rats can differentiate between the long and short panels using this methodology. The experiment ended with two test trials during which the rats were allowed to swim in the arena in the absence of the platforms. The four rectangular panels were the same color as for the training trials for one test, and it was anticipated that rats would spend substantially more time searching for the platforms in the middle of the long than the short panels. For the other test trial, the color of the four panels was opposite to that used for training. The question of interest was whether rats would show a preference for searching near the centers of the walls containing the long panels, when tested with panels of a different color to that used for training.[Fig-anchor fig1]

### Method

#### Subjects

The subjects were 24 male hooded Lister rats supplied by Harlan Olac (Bicester, Oxon, United Kingdom). The rats were approximately 4 months old at the start of the experiment and had received no prior training. They were housed in pairs in a temperature-controlled environment (approximately 20 °C) that was continuously illuminated for 12 hr per day, with lights on at 07:00. Throughout the experiment the rats had free access to food and water. The experiment was performed in accordance with the United Kingdom Animals (Scientific Procedures) Act (1986) and associated guidelines.

#### Apparatus

A white circular pool measuring 200 cm in diameter and 60 cm deep was located 60 cm above the floor in the center of a room (430 cm × 400 cm × 240 cm). The pool was filled with water to a depth of 30 cm and was maintained at a temperature of 24 °C (± 2 °C). The water was made opaque by adding 0.5 L of white opacifier (Opulyn 303B, Dow, United States, catalogue no. 10318500), and it was changed daily. A 2-m diameter white circular ceiling was suspended 1 m above the top edge of the pool. It contained eight 45-W recessed spotlights that were each 22.5 cm in diameter and spaced evenly in a circle with a diameter of 1 m, concentric with the pool. There was a 30-cm diameter hole in the center of the ceiling into which a video camera with a wide-angle lens was fitted. Images from the camera were sent to a monitor in an adjacent room, which also housed recording equipment and a PC with tracking software (Watermaze Software, Edinburgh, United Kingdom). The software was used to record each rat’s swim path and to measure the amount of time spent in different regions of the pool.

A square arena was created from four gray Perspex panels that were each 141 cm long, 60 cm high, and 4 mm thick. Each board was suspended vertically in the pool from bars attached to the upper edge of the outer side of each wall. Fablon panels that were 45 cm high were attached to both sides of each wall. The panels extended from the top of the walls to below the surface of the water. The panels were either matte black or matte white with a width of either 50 cm or 100 cm. The panels were centered horizontally on the wall to which they were attached. For what will be referred to as the black arena, there was a black panel on each of the four walls facing into the pool, with panels of the same length on opposite walls (see Figure 1). The white arena was assembled in the same way, but the four panels were white.

Two circular escape platforms, 10 cm in diameter, were each mounted on a column that rested on the bottom of the pool and resulted in the platform being submerged 2 cm below the surface of the water. The platforms, which had a series of concentric ridges on their surface, were used during all training trials and their centers were situated at a distance of 15 cm from the middle of each long wall. Four identical balls, 10 cm in diameter and covered in colored cartoon characters, were used as landmarks. They were supported by Perspex horizontal rods attached to the middle of the top of each wall. The centers of the landmarks were positioned 15 cm away from the wall to which they were attached. When a landmark was above a platform its center was directly above the center of the platform.

#### Procedure

Rats were trained for four trials in each of 10 sessions, with an intertrial interval of approximately 5 min. A trial started with the rat being lowered gently into the pool facing into a corner, with each corner being used once in every session, in a randomly selected sequence. On reaching a platform the rat was allowed to remain on it for 20 s before being picked up, dried with a towel, and returned to a carrying cage for the remainder of the intertrial interval. Rats that failed to find the platform within 60 s were guided to the platform with a finger that was placed just in front of the rat’s snout. During a trial, the experimenter remained in a small room adjacent to the testing room, where the pool could be observed on a monitor. To ensure subjects relied on the different lengths of the panels of the pool to find a platform, the rectangular arena was rotated between trials by 90, 180, or 270 degrees. This training continued for the remaining four sessions of the experiment, except that the fourth trial of Sessions 11 and 14 consisted of a test in which rats were released from the center of the pool and allowed to swim for 60 s in the absence of the two platforms.

Half the rats were trained in the white arena, and the remainder were trained in the black arena. For six rats in each of these subgroups, the color of the panels was the same as for the training trials for the first test, and the opposite color for the second test. This sequence of test trials was reversed for the remaining rats in each subgroup.

#### Data analysis

The behavior of every rat was observed on the monitor connected to the camera throughout the experiment. During the training trials, the measure of performance was an escape latency, which was defined as the time taken by a rat to climb onto a platform after being released into the pool. The measure during the test trials was the amount of time spent in each of four search zones that were 30 cm in diameter and with centers directly below the centers of the four landmarks. The two zones situated in the middle of the long walls are referred to as the correct zones, while the two zones in the middle of the short walls are referred to as the incorrect zones. In keeping with our previous research (e.g., Kosaki, Austen, & McGregor, 2013), we analyzed the data from the entire 60 s of a test trial. However, when an analysis failed to reveal a significant effect, an additional analysis was conducted using the results from the first 15 s of a test trial.

The analysis of both measures of performance was conducted with analyses of variance (ANOVA) using a rejection criterion of *p* < .05. The reported effect size for ANOVA with more than one factor is partial eta squared (η_p_^2^), while for comparisons between two means it is eta squared (η^2^). For both measures of effect size, 95% confidence intervals (CIs) were computed using the method reported by Steiger (2004).

### Results

**Figure 2 fig2:**
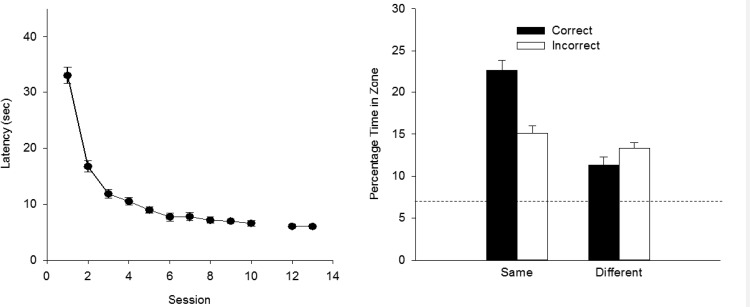
The group mean escape latencies during each complete session of four training trials (left-hand panel), and the group mean percentages of time spent in the correct and incorrect search zones during the two test trials (right-hand panel) for the single group of Experiment 1. Error bars show the standard error. The dashed line indicates the predicted time spent in the test zones if animals searched at random.

For the analysis of escape latencies in each experiment, the results from only the sessions with four training trials were used. A two-way ANOVA of individual mean escape latencies for the first 10 sessions of training, together with those from Sessions 12 and 13 was conducted using the factor of color (whether the panels pasted to the walls were black or white) and session. The analysis revealed a significant effect of session, *F*(11, 232) = 61.46, *MSE* = 22.98, η^2^ = .74, 95% CI [0.67, 0.77], but the effect of color and the interaction were not significant, *F*s < 1. The group mean escape latencies (collapsed across color) are shown in the left-hand panel of Figure 2. In view of the outcome of the ANOVA, the results from the arenas with panels of different color have been combined. This failure to find a statistically significant influence of color was also observed for the test trials of the present experiment. For the sake of clarity of exposition, therefore, this factor has been omitted from the reported analyses and presentation of the data from here on.[Fig-anchor fig2]

The right-hand panel of Figure 2 shows the results from the two test trials. From the left-hand pair of bars it is evident there was a clear preference for searching in front of the 100-cm rather than the 50-cm panels, when testing involved the training panels. This preference, however, was completely absent when testing took place with panels of opposite color to that used for training. A two-way ANOVA, using the factors of search zone (correct or incorrect) and arena (same or different to that used for training) revealed a significant effect of arena, *F*(1, 23) = 61.65, *MSE* = 17.22, η_p_^2^ = .73, 95% CI [0.48, 0.82], a significant effect of zone, *F*(1, 23) = 5.99, *MSE* = 28.42, η_p_^2^ = .21, 95% CI [0.00, 0.45], and a significant interaction, *F*(1, 23) = 14.11, *MSE* = 31.86, η_p_^2^ = .38, 95% CI [0.08, 0.59]. Subsequent tests of simple main effects revealed a significant effect of zone in the arena that was the same as for training, *F*(1, 46) = 19.46, *MSE* = 30.14, η^2^ = .30, 95% CI [0.09, 0.47], but not in the different arena, *F*(1, 46) = 1.10.

In view of the failure to find a significant difference for the entire 60-s test trial with the results for the correct and incorrect search zones, when the panels were different to those used during training, further analysis took place with the results from just the first 15 s of this trial. During the first 15 s of the test trial in the different arena to that used for training, 10.6% of the time was spent in the correct zone and 11.7% was spent in the incorrect zone. This difference was not statistically significant, *F*(1, 23) = 0.21, *MSE* = 66.91. To explore further the failure of rats to discriminate between the correct and incorrect zones during the test trial in the different environment, a Bayesian analysis was conducted using individual percentages of time spent in the correct and incorrect zones (Rouder, Speckman, Sun, Morey, & Iverson, 2009). The analysis tells us if the data favor more the null hypothesis (there being no difference between the percentage time spent in each zone), or the alternative hypothesis (there being a difference between the two sets of percentages). The calculated Bayes factor is the relative probability of the null hypothesis to the alternative hypothesis. A value of 3 would mean that the null hypothesis is three times more likely than the alternative hypothesis given the data and the priors and is suggested by Rouder et al. as the cut off when deciding that results substantially favor the null hypothesis. Analysis supports the null hypothesis when the results for the first 15 s of the trial were considered, Bayes factor = 5.76, but not when the results for the entire 60 s test trial were compared, Bayes factor = 2.83.

In keeping with previous findings (e.g., Kosaki, Jones, et al., 2013), rats readily solved a discrimination in which they had to swim to the middle of long rather than short panels that were attached to the walls of a square pool in order to find an escape platform. There was, however, no hint of this discrimination being sustained when the rats were tested with panels of opposite color to that used for training. As the only cue for solving the discrimination was provided by the length of the panels, this result is at odds with the suggestion that navigation based on geometric cues will be unaffected by a change in the concrete properties of the objects creating the cues (Cheng, 1986; Gallistel, 1990). The principal purpose of the next experiment was to test the generality of the above result. In the present experiment, rats were trained with a discrimination in which the platform was situated beside a long, rather than a short, panel. Experiment 2 explored whether a similar outcome would be found when the submerged platforms were situated near short, but not long panels.

## Experiment 2

The design of the experiment was based on Experiment 1, but the length of the short panels was 25 cm rather than 50 cm. This change was made to take account of the finding by Kosaki, Jones, et al. (2013) that a discrimination in which a hidden goal is placed near a short, but not a long panel, can be solved when the panels are 100 cm and 25 cm, but not when they are 100 cm and 50 cm. There were, therefore, two groups in Experiment 2. Both groups were trained in a square pool with two 100-cm wide panels on one pair of opposite walls and two 25-cm wide panels on the remaining walls. For the short+ group, a submerged platform was situated beside the center of each 25-cm panel, and for the long+ group, the platforms were situated beside the center of the 100-cm panels. The color of the panels was black for half of the rats in each group, and white for the remaining rats. In keeping with the previous experiment, the initial training was followed by two test trials. During these trials the color of the panels was either the same or the opposite of their color for the training trials.

The results from the test trials with the long+ group replicated the findings from Experiment 1, with no hint of a successful transfer of the discrimination on the test trial with panels of different color to that used for training. In contrast, there was evidence of successful transfer when this test took place with the short+ group. One explanation for this unexpected outcome is that animals in the short+ group encoded the length of the panels independently of their color, and used this information to differentiate between the panels during the test trial. There is, however, an alternative explanation for the outcome of the test with the panels of unfamiliar color in the short+ group. When a 25-cm panel was attached to a wall of the arena, then the area to either side of the panel consisted of a region of gray wall of width 58 cm. In contrast, this width was only 20.5 cm when a 100-cm panel was attached to a wall (see Figure 1). Perhaps, therefore, rats in the short+ group used the amount of gray that was exposed on either side of the panels to solve the discrimination, rather than the panels themselves. If this were the case, then changing the color of the panels should not abolish the original discrimination.

In order to choose between the foregoing alternatives, the experiment contained additional training trials, followed by a series of test trials in a gray 90 cm × 180 cm rectangular pool. For these tests, a 25-cm panel was pasted on each of the short walls, and a 100-cm panel was pasted on each of the long walls. As a result, there was an area of gray wall with a width of 32.5 cm to either side of the 25-cm panels and of 40 cm to either side of the 100-cm panels. Two test trials in the rectangle involved panels of the same color as for training. To the extent that the original discrimination trained in the square depends upon learning about the significance of the colored panels, then both groups should devote their time to searching in the vicinity of the panels that were near the platform during the training trials. That is, for both groups, the original discrimination was expected to transfer to panels pasted on the walls of the rectangular pool, when they were the color used for training.

A third test trial, which took place between the two just described, involved panels in the rectangle that were the opposite of the training color. Because the discrimination for the long+ group did not transfer to panels of different color in the square pool, we did not expect it to transfer when a similar test was conducted in the rectangle. Of more interest is the outcome of the equivalent test in the rectangle for the short+ group. If the successful transfer of the discrimination in the square, to panels of different color to that used for training, was based on learning about the significance of the length of the 25-cm panels, then the short+ group should show a preference for the 25-cm over the 100-cm panels irrespective of their color. On the other hand, if the transfer observed in the square pool, when the color of the panels was changed, was due to subjects selecting a wall on the basis of the amount of gray surrounding the panels, then the short+ group should not show a preference for the 25-cm over the 100-cm panels in the rectangle. During this test, the short+ group would be expected to select the wall that displayed a total amount of gray that most closely matched the amount displayed on the wall with the short panel in the square pool. During training, the width of gray either side of a 25-cm panel was 58 cm which, for the tests in the rectangle, is more similar to the 40-cm width of gray beside the 100-cm wide panel on the long wall than the 32.5-cm width of gray beside the 25-cm wide panel on the short wall. On this basis, therefore, subjects should prefer the wall with the long rather than the short panel. In other words, there would now be no reason to expect the short+ discrimination to transfer successfully to the rectangle when the color of the panels was changed.

### Method

#### Subjects

The 36 male rats were of the same stock and from the same supplier as for Experiment 1. They were also housed in the same conditions as for Experiment 1. Prior to the experiment the rats had been deprived to 85% of their free-feeding weights and had received appetitive Pavlovian conditioning in standard test chambers. After the completion of this conditioning they were allowed free access to food for 14 days in their home cages before they were used in the present study. The rats were approximately 4 to 5 months old at the start of the experiment.

#### Apparatus

The apparatus for training and testing in the square pool was the same as for Experiment 1, except that the length of the shorter Fablon panels was 25 cm, rather than 50 cm. A rectangular pool with four gray walls was constructed from two long walls that were 180 cm, and two short walls that were 90 cm. The height of the walls was 60 cm. The walls were suspended from bars in the same manner as for the square pool. Fablon panels, which stretched from the upper edge of the wall to below the surface of the water, were attached to the middle of each wall of the rectangle. The Fablon panels attached to the short walls were 25 cm wide, while those attached to the long walls were 100 cm wide. The panels could be white or black. In keeping with the previous experiment, four landmarks were attached to the upper edge of the middle of each wall of both the square and the rectangle. The landmarks were the same as for the previous experiment, and their centers were 15 cm from the wall to which they were attached.

#### Procedure

The manner of training and testing in the square arena for the first 14 sessions was the same as for Experiment 1. Upon the completion of the second test trial in Session 14, both groups received three cycles of three sessions. The first two sessions of each cycle were standard training sessions that took place in the square pool, and the third session comprised a single test trial in the rectangular pool. The test trials in the first and third cycle involved panels of the same color as that used for training, while for the second cycle the color of the panels for the test trial was opposite to that used for training. Rats were released from the middle of the pool for every test trial. The measures of performance during the training trials in the square, and the test trials in the square and rectangle, were the same as for the previous experiment.

### Results

**Figure 3 fig3:**
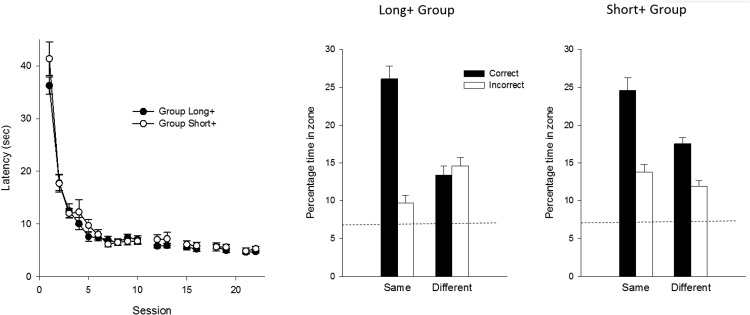
The group mean escape latencies during each complete session of four training trials (left-hand panel) and the group mean percentages of time spent in the correct and incorrect search zones during the test trials in the square arena for the long+ group (center panel) and the short+ group (right-hand panel) of Experiment 2. Error bars show the standard error. The dashed line indicates the predicted time spent in the test zones if animals searched at random.

The left-hand panel of Figure 3 shows the mean escape latencies for the two groups for each of the 18 sessions of training in the square pool. There was a rapid decline in the escape latencies for both groups as training progressed. A two-way ANOVA with the effect of group and session revealed a significant effect of session, *F*(19, 646) = 127.28. *MSE* = 16.82, η_p_^2^ = .79, 95% CI [0.76, 0.80], but the effect of group, *F*(1, 34) = 1.77, and the Group × Session interaction, *F* < 1, were not significant.[Fig-anchor fig3]

The results from the two test trials in the square arena are shown in the center and right-hand panels of Figure 3. It is apparent from the results depicted in the center panel that the results from the long+ group were very similar to its counterpart in Experiment 1. A preference for the long over the short panels was evident when they were of the same color as for training, but not when they were of a different color. The results in the right-hand panel show the results for the short+ group. There was a clear preference for the short over the long panels for the test with panels of the same color as for training, although the extent of this preference was not as marked as for the long+ group. This preference for the short over the long panels was also evident, but to a smaller extent, when the panels were of a different color to that used for training. A three-way ANOVA of individual times spent in the correct and incorrect search zones revealed a significant Group × Panel (same or different to the training color) × Zone interaction, *F*(1, 34) = 9.23, *MSE* = 39.18, η_p_^2^ = .21, 95% CI [0.02, 0.42]. Exploration of this interaction with tests of simple main effects revealed that the short+ group spent significantly more time in the correct than the incorrect search zones during the test with the new panels, *F*(1, 34) = 8.67, *MSE* = 35.17, η^2^ = .20, 95% CI [0.02, 0.41], while this difference was not significant for the long+ group, *F* < 1. Both groups spent significantly more time in the correct than the incorrect search zones during the test with the panels of familiar color, *F*s(1, 68) > 30.82, *MSE* = 35.17, smaller η^2^ = .31, 95% CI [0.14, 0.46]. Moreover, a significant, Group × Zone interaction for the test trial with the familiar panels, *F*(1, 68) = 4.45, *MSE* = 35.17, η_p_^2^ = .06, 95% CI [0.00, 0.19], confirmed that the extent of this preference was significantly greater for the long+ than the short+ group. To return to the original ANOVA, the effect of group, *F*(1, 34) = 1.27, and the two-way interactions involving group, *F*s < 1, were not significant, but the remaining main effects and interactions were significant, *F*s(1, 34) > 30.41, smallest η_p_^2^ = .47, 95% CI [0.21, 0.63].

During the first 15 s of the test trial in the unfamiliar arena the long+ group spent 12.4% of the time in the correct zone, and 14.1% of the time in the incorrect zone. This difference was not statistically significant, *F*(1, 17) = 0.32, *MSE* = 77.53. Further, Bayesian analysis revealed in favor of the null hypothesis for the long+ group when the data for the first 15 s, Bayes factor = 4.79, and for the entire 60 s, Bayes factor = 4.00 were considered.

**Figure 4 fig4:**
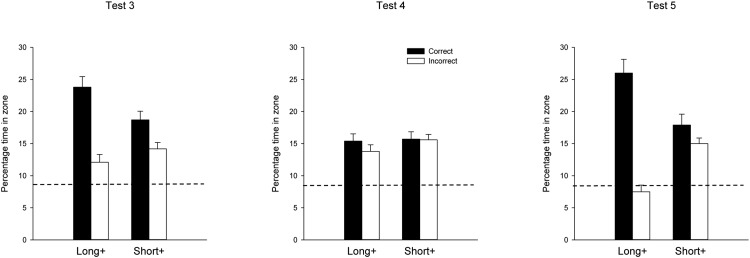
The group mean percentages of time spent in the correct and incorrect search zones during the test trials in the rectangular arena of Experiment 2. The color of the panels on the walls was the same as for training for Tests 3 and 5, and opposite to the training color for Test 4. The dashed line indicates the predicted time spent in the test zones if animals searched at random.

The results from Test Trials 3, 4 and 5, which took place in the rectangle, can be seen in Figure 4. The left- and right-hand panels show the outcome of Test Trials 3 and 5, respectively, for which the color of the panels pasted to the walls was the same as for the training trials. On both occasions, more time was spent in the correct than the incorrect search zones, although this effect was greater for the long+ than the short+ group. When the test involved panels whose color was opposite to that used for training, then a similar amount of time was spent in the correct and incorrect search zones (see center panel of Figure 4). In order to simplify the statistical analysis, the results from the first and third test trials, which took place with familiar panels, were combined. A three-way ANOVA was then conducted with the variables of group, panels (same or different to that for training), and zone, which revealed a significant three-way interaction, *F*(1, 34) = 8.24, *MSE* = 28.87, η_p_^2^ = .20, 95% CI [0.02, 0.40]. Tests of simple effects confirmed that for the test trial with the panels that were different to the training color, the effect of zone was not significant for either the long+ or the short+ group, *F*s < 1. Further tests revealed a significant effect of zone for the combined tests with the long+ group, *F*(1, 34) = 83.00, *MSE* = 26.32, η^2^ = .71, 95% CI [0.51, 0.80], and the short+ group, *F*(1, 34) = 4.76, *MSE* = 26.32, η^2^ = .12, 95% CI [0.00, 0.33], that took place with panels of the same color as during training. Moreover, for the test with the panels that were the same color as for training, there was a significant Group × Zone interaction, *F*(1, 68) = 24.00, *MSE* = 26.32, η_p_^2^ = .26, 95% CI [0.10, 0.41], which again replicates the finding that a long+ short– discrimination is solved more readily by rats than a short+ long– discrimination (Kosaki, Jones, et al., 2013). The remaining findings from the overall ANOVA were as follows. The effect of group, *F* < 1, and the Group × Panels interaction, *F*(1, 34) = 3.75, were not significant, but the remaining main effects and interactions were significant, *F*s(1, 34) > 12.34, smallest η_p_^2^ = .12, 95% CI [0.00, 0.33].[Fig-anchor fig4]

During the first 15 s of Test Trial 4, the long+ group spent the same percentage of time, 11.3, in the correct and incorrect search zones. The short+ group spent 15.8% of the first 15 s in the correct zone, and 12.5% in the incorrect search zone. This difference was not statistically significant, *F*(1, 17) = 1.24, *p* = .28, *MSE* = 96.69. Bayesian analysis found in favor of the null hypothesis for both of these comparisons, Bayes factors >3.14. Additional analyses also found in favor of the null hypothesis when the results for the entire 60 s were analyzed separately for the long+ and the short+ groups, Bayes factors >3.61.

### Discussion

The results from the long+ group confirm that a discrimination based on the length of an object can be completely abolished by changing its color. A similar effect was observed in Experiment 1, but the present experiment extends the findings from that study in two ways. First, the discrimination involved objects with a length of 25 cm and 100 cm, whereas in the previous study they were 50 cm and 100 cm. Second, the experiment has shown that even though the discrimination was maintained when the shape of the test environment was changed from a square to rectangle, it was again abolished when the color of the relevant objects was changed from black to white, or vice versa. It thus appears that the effects of training with a long+ short– discrimination are robust, until the color of the relevant objects is changed.

The main purpose of the present experiment was to examine the effect of changing the color of the objects on which a short+ long– discrimination was based. The results of the test trials in the square indicated that although this manipulation weakened a short+ long– discrimination, it did not abolish it. Rather than demonstrate that in certain circumstances subjects pay heed to the length of an object used for a discrimination, without taking account of the color of the object, the test trials conducted in the rectangular pool point to a more likely explanation for our results. During these test trials, it was apparent that changing the color of the panels on which the discrimination was based eliminated completely the effects of the short+ long– training. As proposed in the introduction to this experiment, the complete disruption of the short+ long– discrimination in the rectangle, but not the square, when the color of the Fablon panels was changed, strongly suggests that this group referred to the amount of gray provided by the walls on which the Fablon panels were pasted. If this cue was used to solve the discrimination in the square, then it would be of no value in the rectangle, where, if anything, it would direct animals toward the incorrect long panels and away from the correct short panels. Taken together, therefore, the results from the present study are entirely consistent with the conclusion drawn from Experiment 1. When solving discriminations based on the lengths of objects, animals place much less emphasis on their abstract properties, such as length, than their concrete characteristics, such as color.

The reliance placed by the short+ group on the gray wall beside the colored panels for solving the discrimination was unexpected. In fact, the results show that the gray wall was not the only cue on which this group relied. During the two tests in the rectangle with panels that were the same color as for training, the short+ group showed a preference for the short over the long panels. This outcome must have been due to the group having learned something about the significance of the black or white panels for finding the goal. The question then arises as why did the short+, but not the long+ group, make use of the additional information provided by the gray walls for finding the platform? One possible answer is that when the large panels indicated where the platform could be found, they overshadowed learning based on the relatively small area of gray wall to either side. Kosaki, Austen, et al. (2013) have shown that stimulus salience influences overshadowing in spatial learning in a manner that is consistent with this proposal.

The results from Experiments 1 and 2 make it clear that navigation based on the geometric properties of an object is severely disrupted when other, more concrete, nongeometric properties of the object are changed, even though its geometric properties remain the same. This pattern of results is inconsistent with the claims by both Cheng (1986) and Gallistel (1990) that the control exerted by the geometric properties of an object will not be affected by a change to their nongeometric properties. It might be argued that these proposals were developed principally with navigation based on cues provided by the shape of the environment in mind, and that for some reason they do not extend to the apparatus used in Experiments 1 and 2. In order to explore this possibility, for the final two experiments, rats were required to find one of two submerged platforms situated in a rectangular pool with four walls of the same color. For Experiment 3, the platforms were situated near the middle of the long, but not the short walls of the arena, and for Experiment 4, the platforms were placed in diagonally opposite corners of rectangular pool with four walls of the same color. They were then tested in the same pool with either walls of the same or opposite color to that used for training.

## Experiment 3

A single group of rats was trained to escape from a rectangular pool that contained an escape platform near the middle of each of its long walls (see the right-hand side of Figure 1). The walls of the arena were all the same color, either black or white. In keeping with the previous experiments a landmark was attached to the top, at the center of each wall. The experiment ended with two types of test trials during which the rats were allowed to swim in the arena in the absence of the platforms. The arena was the same color as that used for training for one test trial, and it was anticipated that during this test rats would spend substantially more time searching for the platforms in the middle of the long than the short walls. For the other test trial, the color of the four walls of the arena was opposite to that used for training, and the question of interest was whether rats would again show a preference for searching near the centers of the long walls.

### Method

#### Subjects

The subjects were 16 male hooded Lister rats that were from the same stock and of approximately the same age as the rats for Experiment 1. The manner of housing was the same as for Experiment 1.

#### Apparatus

The circular pool was the same as for Experiment 1. Two sets of four polyurethane boards, either all black or all white, could be lowered into the pool to create a rectangular arena. The long walls were 180 cm, and the short walls were 90 cm; both had a height of 60 cm. The boards were suspended from aluminum bars, 2 × 2 cm, that rested on the upper edges of the pool. Two circular escape platforms, with the same dimensions as for Experiment 1, were used during all training trials and their centers were situated at a distance of 15 cm from the middle of each long wall. The four landmarks used in the previous experiments were supported by Perspex horizontal rods attached to the middle of the top of each wall. The centers of the landmarks were positioned 15 cm away from the wall to which they were attached. When a landmark was above a platform its center was directly above the center of the platform.

#### Procedure

In each of the nine sessions of the experiment, rats received four trials. All of the trials, except the fourth trial of Session 6 and 8, were training trials that were conducted in the same manner as the training trials for the previous experiments. The remaining two trials were test trials that were conducted in the same manner as for the test trials of the previous experiments. The color of the walls of the rectangle was white for half the rats and black for the remainder. During the first test, for half the rats trained with black walls, and for half trained with white walls, the color of the walls of the rectangle was the same as for the training trials; for the remaining rats, the walls were the opposite color. In the second test the walls were of the opposite color to that for the first test. The measure of performance during the training and test trials was the same as for Experiment 1. Thus, during the test trials in the rectangle, a record was taken of the amount of time that rats spent in circular 30-cm diameter search zones, the centers of which were directly below the centers of the four landmarks. If rats were to search the pool at random, they would spend 8.7% of the test trial in the two correct, or the two incorrect, search zones.

### Results

**Figure 5 fig5:**
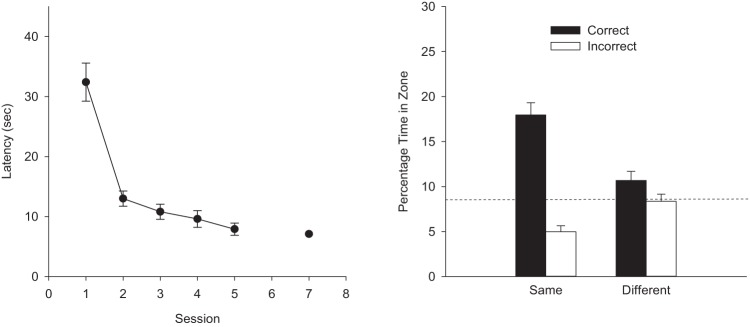
The group mean escape latencies during each complete session of four training trials (left-hand panel), and the group mean percentages of time spent in the correct and incorrect search zones during the two test trials (right-hand panel) for the single group of Experiment 3. Error bars show the standard error. The dashed line indicates the predicted time spent in the test zones if animals searched at random.

The group mean escape latencies for each of the training sessions are shown in the left-hand panel of Figure 5. A one-way ANOVA of individual mean escape latencies for the first five sessions of training, and the additional training that took place in Session 7, revealed a significant effect of session, *F*(5, 75) = 35.84, *MSE* = 40.48, η_p_^2^ = .70, 95% CI [0.57, 0.76].[Fig-anchor fig5]

The right-hand panel of Figure 5 shows the mean percentages of time spent in the two correct search zones combined, and the two incorrect zones combined, for the two test trials. The pair of bars on the left show that considerably more time was spent in the correct than the incorrect search zones when the test took place in an arena that was the same color as that used for training. It is evident from the remaining pair of bars that this difference was disrupted when the color of the walls for the test trial was opposite to that for the training trials. A two-way ANOVA, with the within-subject variables of arena (same vs. different) and zone (correct vs. incorrect) yielded a significant main effect of arena, *F*(1, 15) = 5.66, *MSE* = 10.65, η_p_^2^ = .27, 95% CI [0.00, 0.54], zone, *F*(1, 15) = 41.05, *MSE* = 22.89, η_p_^2^ = .73, 95% CI [0.40, 0.84], and more importantly, an Arena × Zone interaction, *F*(1, 15) = 28.09, *MSE* = 16.16, η_p_^2^ = .65, 95% CI [0.27, 0.79]. Subsequent test of simple effects revealed that significantly more time was spent in the correct than incorrect search zones during the test in the original arena, *F*(1, 15) = 52.87, *MSE* = 25.53, η^2^ = .78, 95% CI [0.47, 0.87], but not the different arena, *F*(1, 15) = 3.23.

### Discussion

A discrimination based on the lengths of the walls of a rectangle was readily apparent when training and testing took place in the same environment. However, when the color of the walls was changed from black to white, or from white to black, for the test trial, then performance on the discrimination was disrupted substantially. Despite the change in methodology, the present results are very similar to those reported in the first two experiments. The results, therefore, support the conclusion drawn from the earlier experiments, that navigation based on geometric cues is adversely affected by a change to the concrete properties of the objects creating those cues. A reexamination of the results shown in the right-hand panel of Figure 5 indicates that it would be premature to conclude from the current experiment that that the change to the appearance of the arena disrupted completely the ability to make use of geometric information provided by its shape. During the test trial in the different environment slightly more time was spent in the correct than the incorrect search zones. Indeed, this difference was statistically significant with a one-tailed test, *t*(15) = 1.80, *p* = .046. The large difference between the test results from the original and the new environment suggests rats initially swam to the platform by referring to information that was not encoded in an abstract fashion. In contrast to the previous experiments, however, it is hard to go one step further and assert with confidence that the solution to the discrimination did not involve a degree of learning based on an abstract representation.

The interpretation of the experiment is clouded, even further, by the fact that the two correct search zones were nearer to each other than the two incorrect search zones. This difference might in some way have biased animals to spending more time searching in the correct than incorrect zones. Although such a bias is unlikely to have resulted in the substantially stronger preference for the correct over the incorrect search zones observed during the test in the familiar test environment, it might have been responsible for the slight preference observed in the different test environment.

## Experiment 4

The experiments thus far have required rats to choose between walls, or panels, of different length by swimming to a platform in the center of one of them. A different task was adopted for the final experiment, in which rats were required to swim to one of two submerged platforms situated in diagonally opposite corners of a rectangular pool. One reason for adopting this task was to test the generality of the results from the previous experiments. If their results are reliable, then even if a rat acquires a strong preference for the geometrically correct over the incorrect corners, it will find it extremely difficult to sustain this preference when the walls of the rectangle are changed from one color to another. A further reason for conducting the experiment was because Cheng’s (1986) proposals concerning the impenetrability of the metric frame to nongeometric information were based on experiments in which rats had to find food in one corner of a rectangle. The possibility thus remains that Cheng’s (1986) and Gallistel’s (1990) proposals are correct, but only in restricted circumstances. In contrast to this possibility, the change to the color of the walls for the test trials resulted in a severe disruption in performance, which indicates that during their original training animals were unable to learn about the significance of the geometric cues independently of their color. An additional test was then conducted in order to test further this conclusion. The test was conducted in a rectangular pool with two adjacent black walls, and two adjacent white walls surrounding the two, diagonally opposite correct corners (see Figure 7). If animals identify the correct corners by referring to geometric cues, then they should treat the two correct corners equally.[Fig-anchor fig7]

### Method

#### Subjects and apparatus

The 24 male rats were from the same stock and of approximately the same age and experience as the rats for Experiment 1. They were housed in the same way as for Experiment 1. The walls creating the rectangular arena were the same as for Experiment 3. In contrast to the previous experiments, landmarks were not attached to the walls of the arena at any stage of the experiment. The two platforms used in Experiment 1 were also used for the present study. When the platforms were placed in the pool, their centers were 25 cm from the nearest corner, on a line that bisected the corner.

#### Procedure

The experiment lasted for 14 sessions with four training trials in every session except for Sessions 8, 10, 12, and 14. These sessions consisted of three training trials followed by a test trial. The four walls of the arena during all training trials were black for half of the rats and white for the remainder. For half of each of these subgroups, a platform was located in the two corners where the long wall of the rectangle was to the left of the short wall; the platforms were situated in the complementary corners of the rectangle for the remaining rats. Rats were released into the pool from the center of a wall, facing into the wall. The orientation of the pool (North, South, East, or West) was changed randomly from trial to trial. The wall from which the rat was released was selected at random from trial to trial. In both cases, there was the constraint that each possible option could be used only once in a session. Rats were released from the middle of the arena for each of the test trials and allowed to swim for 60 s with the platforms removed from the pool. For half the rats, the walls of the rectangle for the test in Session 8 were the same color as for the training trials and in Session 10, they were the opposite color. The reverse of this sequence was used for the remaining rats. The tests in Session 12 and 14 took place in a rectangle for which the four walls were either of the same color as for the training trials, or for which two adjacent walls were black and the remaining two were white. For the test in the rectangle with black and white walls, one geometrically correct corner was constructed from walls that were the same color as the four walls of the training arena, whereas the two walls creating the diagonally opposite correct corner were of the opposite color. The test with the arena that matched the training arena occurred in Session 12 for half the rats that then received the test with the arena composed of black and white walls in Session 14. The opposite sequence was used for the remaining rats.

During each test trial, a record was taken of the percentage of time that rats spent in search zones in each corner of the pool. The zones were 30 cm in diameter and their centers were 25 cm from the corner, on a notional line that bisected the corner. For the purposes of discussion, the two corners that were geometrically identical to those that had housed the platforms during training are referred to as the correct corners, and the other two as the incorrect corners. A record was also taken of the time taken to reach the platform on every training trial. Procedural details that have been omitted were the same as for the previous experiments. If rats were to search the pool at random, they would spend 8.7% of the test trial in any given search zone.

### Results

**Figure 6 fig6:**
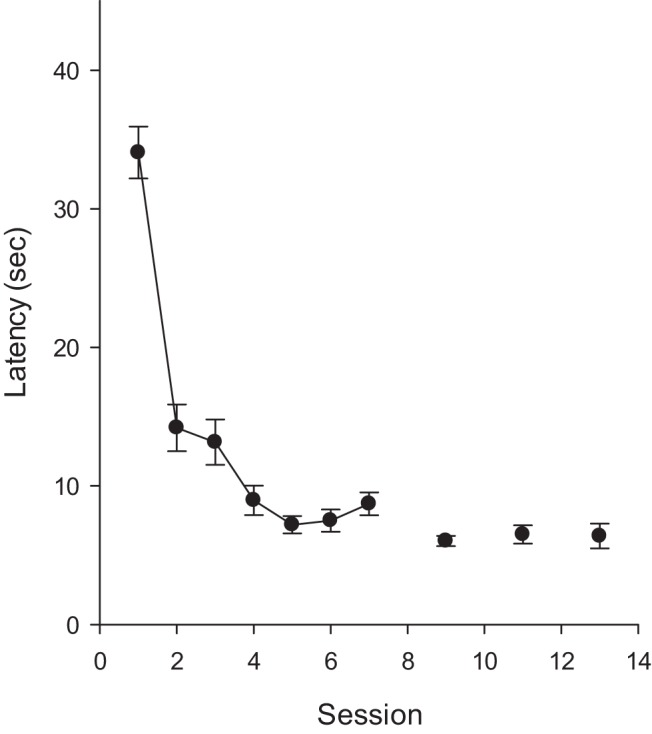
Mean escape latencies during each complete session of four training trials for the single group of Experiment 4. Error bars show the standard error.

The group mean escape latencies for every training session of the experiment are shown in Figure 6. There is no indication that the introduction of the test trials from Session 8 onward had any influence on performance during the training trials. A one-way ANOVA of individual mean escape latencies for each of the 10 sessions of training revealed a significant effect, *F*(9, 207) = 56.82, *MSE* = 30.43, η^2^ = .71, 95% CI [0.64, 0.75].[Fig-anchor fig6]

**Figure 7 fig7:**
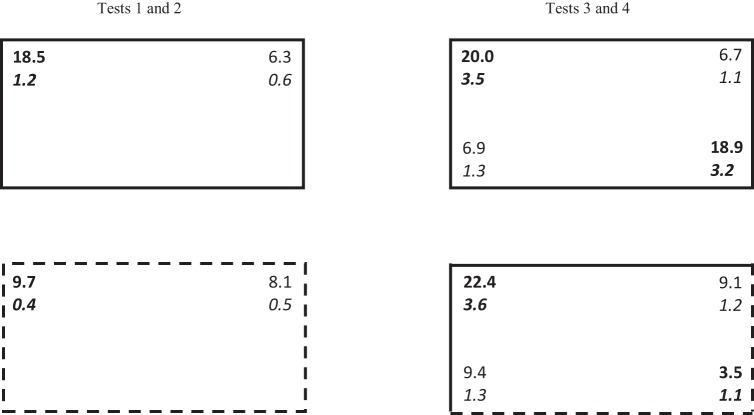
The mean percentage of time spent in the corners of the rectangular arena during the test trials of Experiment 4 (upper number of each pair), and the standard error (lower number of each pair, in italics). Numbers in bold depict the results for the geometrically correct corners. The two panels in the upper row show the results for the test in the arena with walls of the same color as for training. The two lower panels show the results from the test in an arena with either no walls (left-hand panel) or two walls (right-hand panel) of the same color as for training.

The group mean percentages of time spent in the two geometrically correct corners combined, and the two geometrically incorrect corners combined during the first pair of test trials, can be seen in the left-hand column of Figure 7. Note that the data have been normalized so that for all subjects, the geometrically correct corners are regarded as being where a long wall is to the right of a short wall. The results for the geometrically correct corners are highlighted in bold. The upper and lower panels show, respectively, the results from the test in the arena with walls that were the same color, or the opposite color to that used for training. During the test in the original arena, rats spent considerably more time in the correct than the incorrect corners, while during the test in the new arena, the preference for the correct over the incorrect corners was negligible. A two-way ANOVA of individual times spent in the correct and incorrect corners was conducted with the factors of corner (correct or incorrect) and arena (same or different). There was a significant Corner ×Arena interaction, *F*(1, 23) = 50.72, *MSE* = 13.44, η_p_^2^ = .69, 95% CI [0.42, 0.80]. Subsequent tests of simple main effects revealed that significantly more time was spent in the correct than incorrect corners of the original arena, *F*(1, 46) = 106.95, *MSE* = 33.83, η^2^ = .70, 95% CI [0.54, 0.78], but not the new arena, *F*(1, 46) = 1.86. The ANOVA also revealed a significant effect of arena and corner, *F*s(1, 23) > 33.96, smaller η_p_^2^ = .60, 95% CI [0.29, 0.74].

In contrast to the foregoing results, during the first 15 s of the test trial in new arena the percent of time spent in the correct corners, 16.5, was significantly greater than in the incorrect corners, 7.3, *F*(1, 23) = 13.87, *p* < .001, *MSE* = 64.67, η_p_^2^ = .38, 95% CI [0.08, 0.58]. The equivalent values for the test in the original arena were 39.8 for the correct zone and 11.9 for the incorrect zone. Further analysis revealed that significantly more time was spent in the correct corner in the original than in the new arena, *F*(1, 46) = 20.27, *p* < .001, *MSE* = 241.78, η_p_^2^ = .31, 95% CI [0.10, 0.48].

The results from the second pair of test trials, which were given in Sessions 12 and 14, can be seen in the right-hand column of Figure 7, where the time spent in each of the four corners of the test arena is shown. During the test in the arena that was the same as that for training (upper panel), substantially more time was again spent in the correct than the incorrect corners. When two of the walls were of a different color to that for training (lower panel), then there was a clear preference for the geometrically correct corner whose walls were the same color as during training. Moreover, the amount of time spent in this corner was similar to the time spent in the geometrically correct corners during the accompanying test in the original arena. Rather little time was spent in the two geometrically incorrect corners, and the least amount of time was spent in the geometrically correct corner that was the opposite color to that for training.

A three-way ANOVA with the factors of corner, arena, and side (whether the correct and incorrect corners were situated at the ends of the long wall to the north or the south of the pool) revealed a significant three-way interaction, *F*(1, 23) = 5.45, *MSE* = 177.29, η_p_^2^ = .19, 95% CI [0.00, 0.43]. Subsequent tests of simple main effects revealed a significant preference for the correct over the incorrect corner on both sides of the rectangle in the test in the original arena, *F*s(1, 92) > 11.75, *p*s < .001, *MSE* = 146.91, smaller η^2^ = .11, 95% CI [0.02, 0.24]. A similar preference was also seen in the arena with black and white walls, but only when the correct corner was created by walls of the same color as for training, *F*(1, 92) = 14.50, *MSE* = 146.91, η^2^ = .14, 95% CI [0.03, 0.27]. Significantly less time was spent in the remaining geometrically correct corner in this arena when compared either with the other geometrically correct corner in the same arena, *F*(1, 92) = 22.06, *MSE* = 195.35, η^2^ = .19, 95% CI [0.07, 0.33], or to the equivalent corner in the training arena, *F*(1, 92) = 26.23, *MSE* = 108.93, η^2^ = .22, 95% CI [0.09, 0.36]. Although less time was spent in the correct corner created by the new walls than in the incorrect corner at the other end of the long wall, this difference was not significant, *F*(1, 92) = 2.86, *p* = .094. To return to the overall ANOVA, the three main effects and all the two-way interactions were significant, *F*s(1, 23) > 5.43, smallest η_p_^2^ = .19, 95% CI [0.00, 0.43].

### Discussion

The results from the entire 60-s test trial indicate that rats paid very little heed to the geometric cues provided by the shape of the environment during the test in the arena with walls of a new color. Furthermore, in the rectangle with two white walls and two black walls, rats spent less time in the geometrically correct corner with walls of opposite color to that for training than in any other corner. In keeping with the previous experiments, the results suggest that rats find it difficult to refer to the geometric properties of an object, when the color of that object is changed.

The conclusion just reached is tempered by the finding that during the first 15 s of the test in the arena with new walls, there was a modest but statistically significant preference for the correct over the incorrect corners. It is not easy to know how to explain this outcome. One possibility is that learning about the position of the platform with reference to geometric cues was not entirely disrupted by changing the color of the walls of the pool. Another possibility is that this manipulation disrupted completely the control exerted by geometric cues, and that the presence of some other cue was responsible for the modest preference for the correct over incorrect corners that was observed in the first 15 s of the first test trial. The rectangular arena was surrounded by a circular curtain. If a rat was to look beyond the upper edges of the walls while swimming it would notice that the curtain fell nearer to the middle of some walls (the short walls), than other walls (the long walls). Rather than discriminate between the walls in terms of their length, therefore, rats may have used the overall proximity of the walls to the curtain. Of course this strategy would still be effective after the color of the walls had been changed and may have been responsible for the modest preference shown for the correct corners. This possibility, notwithstanding, we can conclude that rats find it difficult to respond on the basis of geometric cues, when the color of the objects creating those cues is changed. Whether there is some residual influence of these cues after such a change has been effected remains to be determined. The results from Experiments 1 and 2 suggest not, while the results from the present experiment suggest that if there is an influence, it is slight.

## General Discussion

There is no doubt that animals can learn to navigate with reference to the length of objects. In the absence of this ability, for example, they would be unable to head directly for a goal hidden consistently in one corner of a rectangular arena. The main purpose of the present article has been to determine how animals represent this property of an object. In particular, we have explored the suggestion of both Cheng (1986) and Gallistel (1990) that the length of an object is encoded in an abstract manner that permits it to be used to identify where a goal is situated independently of its nongeometric, concrete properties. A straightforward implication of this proposal is that once an animal has identified the location of a goal, with reference to the geometric properties of an object, then any change to other properties of the object should not affect navigation. The results from all four experiments challenge this proposal. Experiments 1 and 2 revealed that a discrimination based on panels of different length was abolished by changing their color, and Experiments 3 and 4 showed that changing the color of a rectangular arena very seriously affected how rats made use of it to find an escape platform. Moreover, in the discussion to Experiment 4 it was suggested that the modest preference for the correct corners during the test in the new arena might have been due to the presence of cues that remained unchanged from the training trials.

Hitherto, the majority of experiments investigating the role of geometric information in navigation by animals have investigate the extent to which the use of such information is influenced by the presence of nongeometric cues that also provide information about where the goal is situated. For example, Pearce, Graham, Good, Jones, and McGregor (2006) demonstrated that when the color of the walls of a rectangular arena could be used to find a goal, then learning about the position of the goal relative to the geometric cues was greatly restricted. (see Cheng, 2008, and Pearce, 2009, for reviews). The present experiments go beyond these results by showing for the first time that the control exerted by geometric cues over searching for a goal is severely disrupted by a change to nongeometric cues, even when the latter are of no help for indicating where the goal can be found.

One interpretation of our findings can be developed in terms of generalization decrement. Once an animal has been trained to find a goal in front of a long, rather than a short black panel, it might identify the position of the goal with reference to an abstract representation of their length. The modification to the test environment brought about by a change to the color of the panels might then disrupt the control exerted by geometric information. A problem with this account is that the lack of evidence showing that animals make use of abstract information about the length of objects. Moreover, this interpretation goes directly against arguments put forward by Gallistel (1990, pp. 211–212) that were summarized in the introduction.

The results from Experiments 2 and 3 provide rather different reasons for questioning whether the absence of transfer of a length discrimination from objects of one color to another is due to generalization decrement. We demonstrated in Experiment 2 that a discrimination between two panels of different length remained intact when the panels were moved from the square training arena to the rectangular test arena. This finding suggests that the outcome of a length discrimination is not as sensitive to the effects of generalization decrement as the foregoing account implies. Turning now to Experiment 4, after being trained to find a platform in two diagonally opposite corners of a rectangle, rats were tested in the same arena but with the walls creating one of the correct corners changed to the opposite color to that used for training. Despite this change to half of the arena, there was no hint of a generalization decrement, as far as performance in the corner surrounded by walls of the original color was concerned. There was, however, a marked loss of interest in the other correct corner, where the color of the walls was opposite to that used for training. If animals made use of abstract metric information to identify the correct corners, then during this test it is not clear why such information was readily used to identify one of the correct corners, but not the other.

In view of the foregoing discussion, an obvious implication to draw from the present experiments is that, when learning about the position of a goal, rats place little or no importance on abstract information about the length of objects. The question then arises as to how they were able to identify accurately where a platform could be found during the training stages of the above experiments. One possible answer is that when rats reach their goal they take a mental snapshot of their surroundings (Collett & Collett, 2002; Sheynikhovich, Chavarriaga, Strösslin, Arleo, & Gerstner, 2009; Stürzl, Cheung, Cheng, & Zeil, 2008). On being returned to the pool, they are then assumed to move in such a way that the disparity between their current view and the mental snapshot is progressively reduced. When the disparity has been reduced to zero, then the subject will have reached its goal. Superficially, at least, this account is able to explain our basic findings. If a rat takes a mental snapshot when it comes across a platform in one corner of a black rectangular pool then, when it is placed in a similar rectangle with white walls, the change in color of the walls will make it impossible for the animals to swim to a location where its current view matches the mental snapshot. A similar problem would be expected if the rat is trained to swim to the middle of the longer of two black panels (or walls), and then tested with two white panels (or walls).

Closer inspection, however, reveals two potential problems for such a template matching account of our findings. One problem is posed by the test trial in Experiment 4, in which rats were placed in an arena with two white walls and two black walls. The test revealed that the amount of time spent in the correct corner that was constructed from walls of the same color as the training arena was unaffected by the change in color to the opposite walls of the arena. According to at least one account, when a mental snapshot is taken, it encompasses the entire 360° view available to the subject (e.g., Stürzl et al., 2008). On this basis, it would be expected that the manipulation just described, by ensuring that the snapshot no longer matched fully any view of the arena, would make it harder for the animal to identify as correct the corner where the walls were the same color as for training. Perhaps the failure to confirm this prediction occurred because mental snapshots are restricted to more local views than the panorama envisaged by Stürzl et al. (2008).

The second problem is posed by the results of Experiment 2, where it was found that the discrimination between the long and short panels was more marked when the platforms were situated in front of the long panels rather than in front of the short panels (for a similar result, see Kosaki, Jones, et al., 2013). If the location of the goal is identified by means of a mental snapshot, then it is not clear why a snapshot taken in front of a large panel should be a more effective guide for reaching the goal than one taken in front of a short panel.

A rather different explanation for how rats are able to find a goal in the present experiments is based on principles of associative learning (see Kosaki, Jones, et al., 2013). Consider the discrimination in Experiment 1 based on black panels. Rats might associate large and small black panels, respectively, with the presence and absence of reward. Once formed, these associations would then guide the rats toward the large and away from the small panels. Moreover, rather than represent the panels in terms of their length, rats may represent them in terms of the overall stimulation they provide or, for the present example, the number of receptors for blackness they excite. Changing the color of the panels from black to white would then weaken responding through the removal of critically important excitatory cues and result in little or no transfer from the training to the test trials. There is no reason why these principles should not extend to a rectangle constructed, say, from four black walls. The different lengths of the walls would be represented by the different number of blackness receptors they excite. The goal could then be found by identifying the correct location as the one at a particular end of a wall that excited a particular number of blackness receptors. Changing the color of the walls from black to white would, again, eliminate the preference for correct over incorrect corners.

The superior discrimination when the platform was situated in front of the large rather than in front of the small panels in Experiment 2 can also be explained by associative learning principles. Once again, we shall assume color of the panels was black. Following proposals by Mackintosh (1974, see also Perkins, 1953; Logan, 1954), Kosaki, Jones, et al. (2013) suggested that discriminations based on stimulus intensity, such as the amount of blackness, involve three values from the same dimension. Thus S_small_ and S_large_ might represent small and large amounts of blackness, respectively, and S_o_ might represent the absence of any black stimulation. During either a S_small_+ S_large_–, or a S_large_+ S_small_– discrimination, S_o_ will inevitably gain inhibitory strength as the animals discovers that regions of the pool without a black panel do not contain a platform. This inhibition can then be expected to generalize to both S_small,_ and S_large_, but to a greater extent to the former than the latter, and result in the S_small_– S_large_+ discrimination progressing more readily than S_small_+ S_large_ –.

Although this account can explain all the findings we have described, it is not too hard to identify a potential problem with it. Rats might be trained in the manner used for Experiment 1, but with four rectangular panels that were all of the same size, oriented either horizontally or vertically, and with the platform in front of panels of a particular orientation. It is likely that the discrimination would be solved, but because each panel will provide the same amount of black stimulation, the implication of the above analysis is that the discrimination would be impossible. Of course, a template matching account would find it easy to explain the anticipated result, and perhaps a combination of associative learning and template matching principles is required if a satisfactory explanation for our results is to be developed. For instance, excitatory and inhibitory associations might develop to patterns of stimulation akin to templates, and stimulus generalization based on the similarity between patterns would determine the strength of approach and avoidance to each of them (e.g., Pearce, 1994).

A fundamental strength of both the template-matching and associative-learning account for the present results is that neither of them assumes animals represent the geometric properties of objects in an abstract manner that is independent of the nongeometric, concrete properties of the objects. They are thus compatible with the findings from all four experiments and merit further evaluation and development if our understanding of how spatial learning based on geometric cues takes place. At the same time, the results lend scant support to any theoretical analysis that holds animals acquire abstract information about the geometric properties of their environment.
